# X-ray Absorption and Magnetic Circular Dichroism in CVD Grown Carbon Nanotubes

**DOI:** 10.3390/ma12071073

**Published:** 2019-04-01

**Authors:** Stefano Bellucci, Antonino Cataldo, Alberto Tagliaferro, Mauro Giorcelli, Federico Micciulla

**Affiliations:** 1 INFN-Laboratori Nazionali di Frascati, via E. Fermi 40, 00044 Frascati, Italy; stefano.bellucci@lnf.infn.it (S.B.); antonino.cataldo@lnf.infn.it (A.C.); 2 Dipartimento di Neuroscienze, Imaging e Scienze Cliniche, via dei Vestini, 33 66100 Chieti, Italy; 3 Politecnico di Torino, Dipartimento di Scienza dei Materiali e Ingegneria Chimica, Università degi Studi di Chieti e Pescara “G.D’Annunzio”, Corso Duca degli Abruzzi 24, I-10129 Torino, Italy; alberto.tagliaferro@polito.it (A.T.); mauro.giorcelli@polito.it (M.G.)

**Keywords:** magnetic dichroism, carbon nanotubes, synchrotron radiation spectroscopy

## Abstract

Nowadays, a deep knowledge of procedures of synthesis of nanostructured materials plays an important role in achieving nano-materials with accurate and wanted properties and performances. Carbon-based nanostructured materials continue to attract a huge amount of research efforts, because of their wide-ranging properties. Using X-ray absorption (XAS) and X-ray magnetic circular dichroism (XMCD) spectroscopy in the soft X-ray regime, by the synchrotron radiation, we studied the L3,2 absorption edges of iron (Fe) nanoparticles, when they are embedded in oriented Multi Wall Carbon Nanotube (MWCNTs) layers grown by thermal Chemical Vapor Deposition (CVD) technique catalyzed by this transition metal. This could allow us to understand the valence state and role of catalysts and thus their electronic and magnetic structures. It is important to note that the control of the size of these tethered nanoparticles is of primary importance for the purpose of tailoring the physical and chemical properties of these hierarchical materials. The MWCNTs samples used in XAS and XMCD measurements were synthesized by the CVD technique. The actual measurements were carried out by the group NEXT of the INFN- LNF with the logistic experimental support of the INFM-CNR and the Synchrotron Elettra Trieste.

## 1. Introduction

The study of carbon nanotubes (CNTs) [1] has opened up research in many disciplines, as these materials possess many unique physical properties with novel applications in various fields [2]. It has been shown that these new materials can be tailored to have many interesting properties: Metallic, semiconducting, and magnetic. Among the number of methods to synthesize CNTs, the chemical vapor deposition method is used to prepare selectively single, double, and multi-walled CNTs [3]. This method uses mainly 3d transition metals (TM) Fe, Ni, and Co as catalysts, which come into contact with tube walls and significantly influence the transport, magnetic, and electronic properties. Synchrotron radiation source-based studies, such as high energy electron spectroscopies are likely to provide a better insight in understanding the magnetic and electronic structure of these materials. The X-ray absorption and magnetic circular dichroism in the soft X-ray regime attract considerable interest, owing to the possibility of extracting microscopic information about spin and orbital momenta with element specificity [4].

In optics, the term “dichroism” refers to changes in the absorption of polarized light on passing through a material in two different directions. Since materials typically absorb one color of white light preferentially, the material appears with two different colors for the two light directions—it is di- (two-) chroic (colored) [5]. Actually, the term dichroism is used more generally to reflect the dependence of photon absorption of a material on polarization [6]. The origin of the dichroism effect can be anisotropies in the charge or the spin in the material. In this case, it is possible to speak of magnetic dichroism [7]. A brief introduction to the principles and applications of magnetic dichroism techniques in the X-ray region, the X-Ray Magnetic Circular Dichroism (XMCD) and X-Ray Magnetic Linear Dichroism (XMLD) techniques can be found in reference [8]. It also gives information on the chemical environment of the atoms (NEXAFS Spectroscopy) and their magnetic state. Core electrons are excited in the absorption process into empty states above the Fermi energy and thereby probe the electronic and magnetic properties of the empty valence levels. In the following, we are concerned with the spectra of the magnetic 3d transition metal elements Fe, Co, and Ni. Their magnetic properties are largely determined by the 3d valence electrons. Since X-ray absorption spectra are driven by dipole selection rules the d-shell properties are best probed by the L-edge absorption studies (2p to 3d transitions) [8]. The metals are usually ferromagnetic, and their magnetic properties are best studied with XMCD spectroscopy, while the oxides are usually antiferromagnetic and are studied with XMLD spectroscopy [8]. 

The motivation for the present investigation stems from recent findings indicating that CNTs can be magnetized when placed in contact with a magnetic material. It is also observed that the basic electrical properties of semiconducting CNTs change when they are placed inside a magnetic field. In fact, semiconducting nanotubes can become metallic when immersed in a very strong magnetic field, while metallic tubes can be made semiconducting by applying a (even small) magnetic field parallel to the tube axis. These exotic magnetic effects are related to the modulation of the electronic wavefunction along the tube circumference by the Aharonov–Bohm phase [9].

Carbon nanotubes can be either paramagnetic or diamagnetic depending on their chirality. This behavior, with the corresponding change in physical properties, could be important in many novel applications as nano-devices. For example, the selectivity in electrical conductance of a sample made of CNTs of a specific character (i.e., predominantly metallic) could enhance the electromagnetic screening efficiency of a nanocomposite for aerospace applications [10]. 

In addition, a nano-interconnection for Information Commmunication Technology (ICT) applications would depend crucially on such a selective skill, insofar as the performance of the corresponding device is concerned [11]. Magnetism can be induced in carbon structures by defects such as vacancies or impurities, too. In the absence of defects, all bonding electrons are paired in π bonds. However, defects that delocalize one of the pair bonds induce excess spin polarization. This can lead to ferromagnetism (FM) when the defects population is sufficiently dense [12]. However, it is important to underline how the origin of magnetism in carbon materials is still controversial [13].

## 2. Materials and Methods

### 2.1. Preparation of Samples by CVD Synthesis

The Chemical Vapor Deposition (CVD) technique involves the decomposition of a gaseous or liquid compound of carbon, catalyzed by metallic nanoparticles, which also serve as nucleation sites for the initiation of carbon-nanotube growth at temperatures of 600–1000 °C [14]. CVD is a heterogeneous catalysis process that can easily be scaled up to industrial production levels. It has in fact become the most important commercial method for single-walled CNTs (SWNTs) and Multi Wall Carbon Nanotube (MWCNTs) production [15,16,17,18]. However, the CVD growth of CNTs requires the presence of transition metals like Fe, Ni, or Co as catalysts. It exploits their possibility to be transformed during the reaction into metastable carbides and their high affinity to form solid solution with carbon, allowing its diffusion through the metal particles to form ordered carbon nano-structures (nanotubes or nanofibers) [19,20].

A deep knowledge and a high control of CVD growth parameters are required to obtain SWCNTs or MWCNTs with precise characteristics: Diameter, length, number of walls, and chirality. Different amounts of the most widely used catalytic materials, Ni and Fe, of nanoscale particle size significantly influence the chirality distribution of as-grown SWCNTs. Recently, bimetallic catalysts such as CoMo and FeCo have gained relevance. Micro-characterization of the bimetallic nano-catalysts suggests an intimate relationship between the catalyst structure and the final nanotube chirality [21]. 

In this study, we focused on MWCNTs. The latter have been synthesized on p-type (Boron doped; resist: 40–100 ohm) Si (100) substrate by the CVD process. Using gaseous Ferrocene [Fe(C5H5)2] as a catalyst and Camphor [C10H16O] as the main carbon source [22] (recall that ferrocene provides not only the iron catalyst but also part of the carbon for nanotube growth). Two different ratios in volume between Ferrocene/Camphor were employed: 1:30 and 1:20 (Table 1). In both cases a suitable selection of growth parameters allowed us to obtain vertically aligned CNTs [23]. 

The growth mechanism can be outlined as follows. Ferrocene liberates metal nanoparticles in situ, which catalyzed the hydrocarbon decomposition more efficiently. MWCNTs diameters were correlated with the catalyst particle size. Previous analysis suggested that the growth mechanism was a tip-growth one, in which the catalyst particles moved far from the substrate, leaving behind the CNT and remaining encapsulated close to their tips [24] 

**Figure 1:**
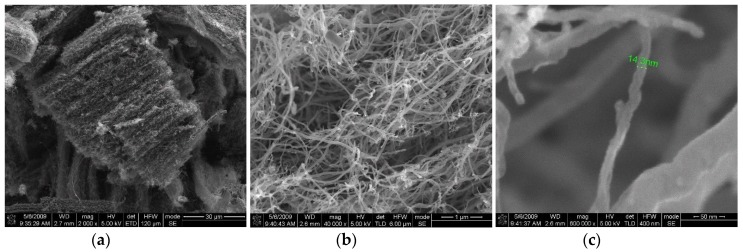
Images of carbon nanotubes (CNTs) grown using high iron amount (1:20 sample).

**Figure 2:**
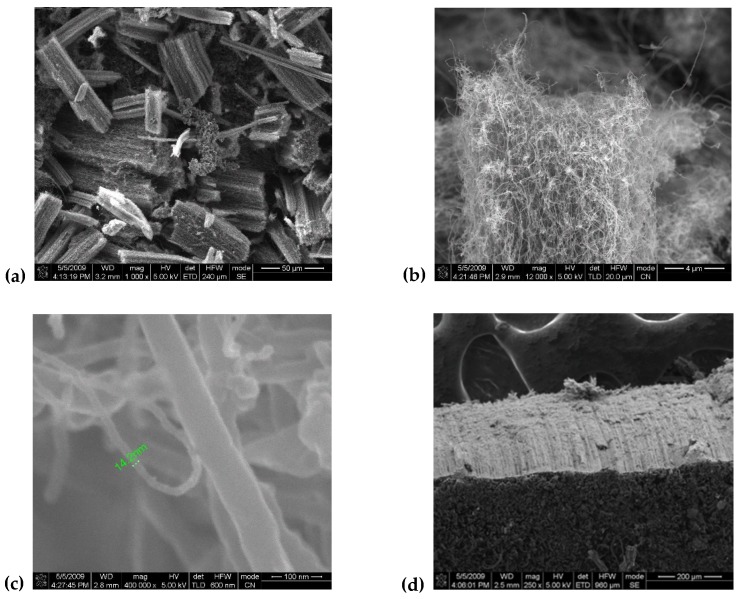
Images of CNTs grown using low iron amount (1:30 sample).

We characterized all CNTs samples by scanning electron microscope (SEM), using an ultra-high-resolution Field Emission—SEM, Nova™ NanoSEM 630 and high resolution transmission electron microscopy (HRTEM), FEI Titan 80–300. In Figure 1 and Figure 2 the images corresponding to the different parameters given in Table 1 are shown. The vertical alignment of the nanotubes in the samples is traceable (see Figure 2d), see also Ref. [23]). The size of the nanotubes is also quite homogeneous, i.e., about 14 nm for both types of samples corresponding to the two sets of parameters in Table 1.

### 2.2. The Experimental Apparatus for Magnetic Dichroism Investigation

The experiments were carried out at Elettra, Trieste (Italy). The B.A.C.H. beamline (Beamline for Advanced diCHroism) is an undulator beamline of the Istituto Officina dei Materiali-Consiglio Nazionale delle Ricerche, which operates at Elettra, in close collaboration with Sincrotrone Trieste S.C.p.A. The beamline offers a multi-technique approach for the investigation of the electronic, chemical, structural, magnetic, and dynamical properties of solid surfaces, interfaces, thin films and solid samples in the UV-soft X-ray photon energy range (35–1600 eV) with selectable light polarization (linear horizontal and vertical, circular), high resolving power (20,000–6000), and time resolution (70 ps) [25].

### 2.3. The Experimental Measurements

The X-ray absorption and XMCD at K-C, Fe L3,2-edges of these CNTs have been measured by the Total Electron Yield (TEY) method. These measurements helped us to identify the electronic and magnetic structure of CNTs grown with iron as a catalyst, and hence its spectroscopic magnetic behavior.

The experimental results we will discuss below can be correlated with ab initio theoretical calculations. Using such studies, we can explore the relationship between the electronic properties of CNTs, the role of catalysts and their chemical state, magnetic structure, and the number of walls. Work on this issue is in progress and will be reported elsewhere.

In order to carry out suitable blank measurements needed to establish the comparison levels against which to evaluate the results from the samples, an iron standard was used.

**Figure 3:**
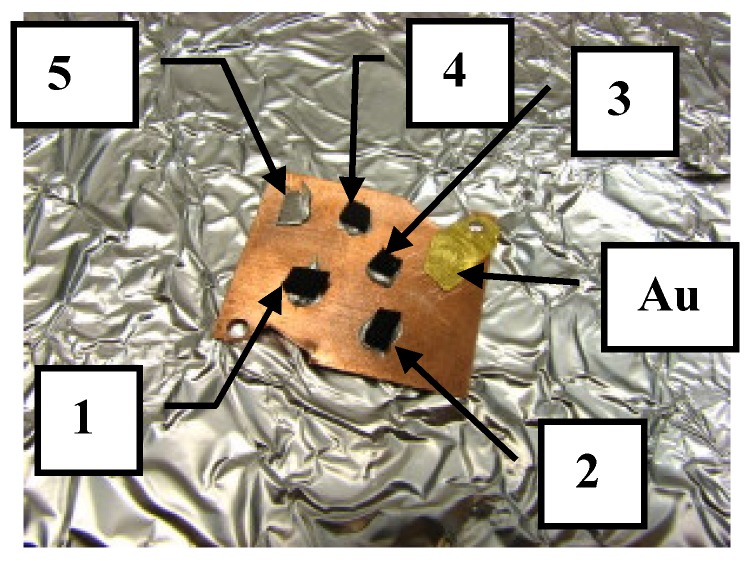
Images of CNTS-Fe copper holder with samples on top: 1—CNTSs-20Fe-s; 2—CNTSs-20Fe-r; 3—CNTSs-30Fe-r; 4—CNTSs-30Fe-s; 5—Fe-Foil-99% pure.

The investigation started from the CNTs-Fe samples (FCCF158 and FCCF111). These two samples have the appearance of a mat whose dimensions are about 1 cm (wide) × 2 cm (long) × 2 mm (thick). They were grown on a Silicon substrate and subsequently detached from it. One of the faces, namely the one in contact with the substrate prior to the detachment, appears smooth, while the other appears rough at visual analysis. Two pieces whose extension was about 0.5 × 0.5 cm^2^ each were cut off from each sample. Having two chips for each sample gave us the chance to analyse both the smooth and the rough surfaces. Samples were stuck on the copper holder by using conductive epoxy glue. In Figure 3, for the sake of clarity and illustrative purposes, we report a picture of the samples mounted on the holder. Along with the four chips, indicated as CNTS-20Fe-s, CNTS-20Fe-r, CNTS-30Fe-s, and CNTS-30Fe-r (where s and r stand for smooth and rough surfaces, respectively), a small piece of iron standard foil with 99% purity was stuck by the same glue on the sample copper holder (position 5, Figure 3).

A piece of golden foil was also added to the sample holder thus that the values of the energy in the X-ray spectra could be calibrated against the energy of the 4d electrons of gold whose value is known with great accuracy.

The four samples and the iron foil were magnetized using a 0.5 Tesla permanent magnet thus that the direction of the induced magnetization was perpendicular to the holder surface and parallel to the tube axis. The same facet of the magnet was placed at about 1 mm from the surface of each sample to be magnetized and made it slightly oscillate around such position for 1 min. The sample holder was then (i) inserted into the external vacuum chamber (whose pressure reaches the order of 10–7 torr) (ii) pushed into the pre-vacuum chamber (whose pressure reaches the order of 10–9 torr) and eventually (iii) into the measurement vacuum chamber (whose pressure reaches the order of 10–9–10–10 torr). 

The energy values of the beam were calibrated in order to tune the beam line on the L3,2 iron edges. The X-ray absorption (XAS) measurements were carried on by revealing the TEY. The calibrations determined the frequency range to be scanned and the aperture of the entrance and exit slits of the monochromator, as well as the range of the Keithley current meters. All these calibrations, carried out remotely by Lab View software, were aimed at achieving the highest signal to noise ratio. Four different scans were planned per each sample in order to obtain the best possible conditions for the measurements:

1. Horizontal polarization—normal incidence (X-ray beam perpendicular to the sample surface—corresponding to 88° on the angular scale of the manipulator)
2. Right circular polarization (positive phase on the control software)—normal incidence
3. Left circular polarization (negative phase on the control software)—normal incidence
4. Linear polarization—oblique incidence (angle of 60° between the X-ray beam and the normal to the sample surface—corresponding to 148° on the angular scale of the manipulator)

## 3. Results

**Figure 4:**
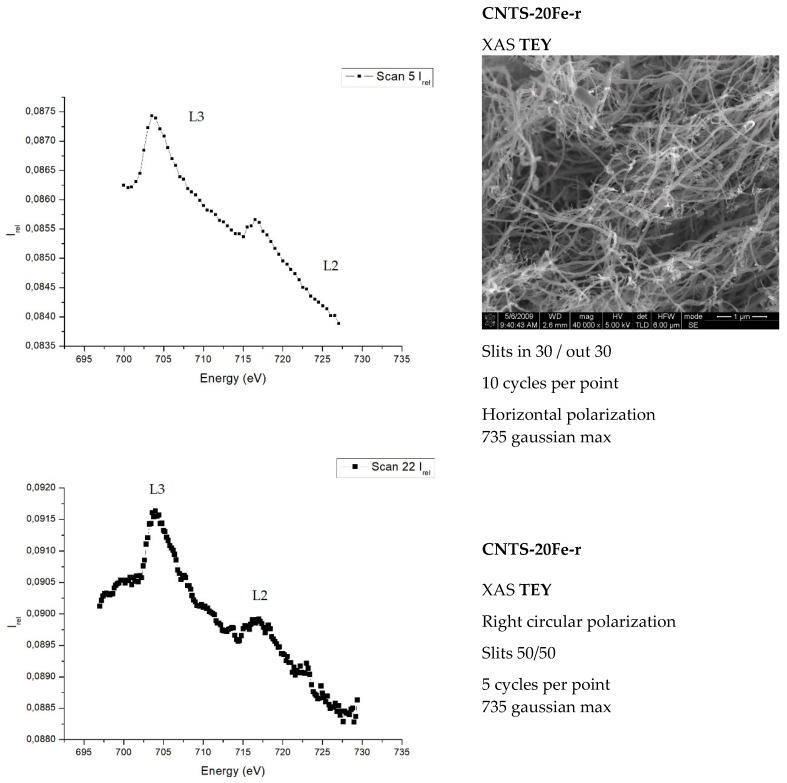
The two graphs above show the response of the CNTS-20Fe-r sample (r = stands for rough surface). The same typical response of the L3,2 iron edge was obtained from the rough surface of the CNTs-30Fe-r (sample which we expect to have a lower iron content); on y axis, the intensity is reported, it is in arbitrary units.

**Figure 5:**
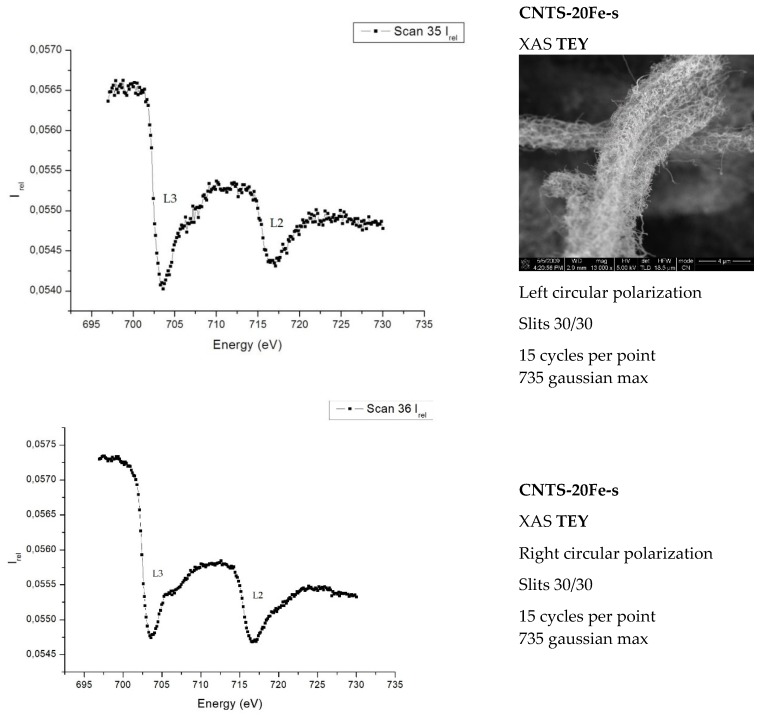
The two graphs above show the response of the CNTS-20Fe-s sample.

The TEY measurements carried out on the two morphologically different surfaces produced different results. Measurements carried out on the rough surface of the MWCNT forest show (see Figure 4) the typical upward bumps, related to iron L3,2 edges, on a decreasing background. In contrast, when the scan with linear polarization and oblique incidence was performed on the smooth surface of the MWCNT forest, quite surprisingly, the peaks showed up with an inversion of their intensity with respect to the decreasing background (see Figure 5). This behavior suggests the existence of an angle or a range of angles over which the inversion of the iron peaks would take place. In both Figure 4 and Figure 5 the vertical axes show transmitted beam intensities in arbitrary units. The XAS beam parameters and other details used to perform each experiment are given in the boxes on the right of each picture.

The width of the slits which allow for the signal to reach the samples, as input, and then the detector, as output, were chosen and the cycles carried out in order to achieve a better ratio between the signal and noise for each sample.

Figure 4 shows that the spectra of the samples, compared to the Fe-metal spectrum (not reported) have

(i) A lower L3/L2 peak-intensities ratio;
(ii) An increase of the width of the peaks.

This indicates the presence of Fe-O and Fe-C compounds [8,26].

In summary, dichroism is appearing in both Figure 4 and Figure 5, although the interesting feature of the peak’s inversion arises only in the experiments involving the rough face of the samples. From Figure 4 and Figure 5, we can draw an interesting conclusion: The signal of the Fe is present in the “rough” part (upper) and not in the “smooth” part (lower, i.e., the one in contact with the silicon wafer). As we will discuss in the conclusions at the end of the paper, the tip growth of the CNTs of the sample occurring bottom-up can help us to understand the peculiarity in the catalyst (Fe) signal; on y axis the intensity is reported, and it is in arbitrary units.

**Figure 6:**
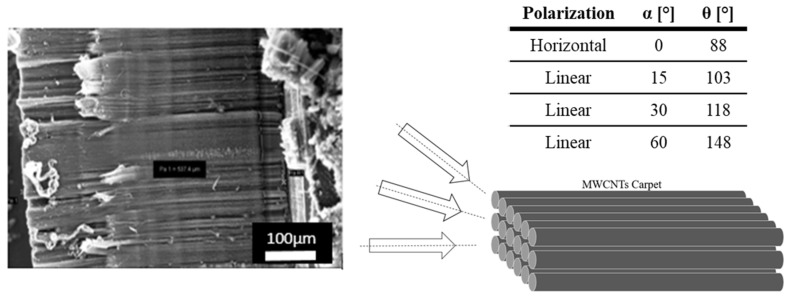
Image of the aligned Multi Wall Carbon Nanotube (MWCNTs) and the beam incident angles used.

In the following we explore a possible explanation of this peculiar behavior by investigating further scans at decreasing angles. The signal was upward at 60° and 30° (118° on the manipulator), while it was inverted at 15° degrees (103° degrees on the manipulator), see Figure 6.

The TEY depends on the angle between the surface normal and the axis of the microscope optics. Therefore, the peak intensity is sensitive to the specific crystallographic orientation of each iron nanoparticle [27] and of the external shell of CNTSs [28] with respect to the substrate. 

**Figure 7:**
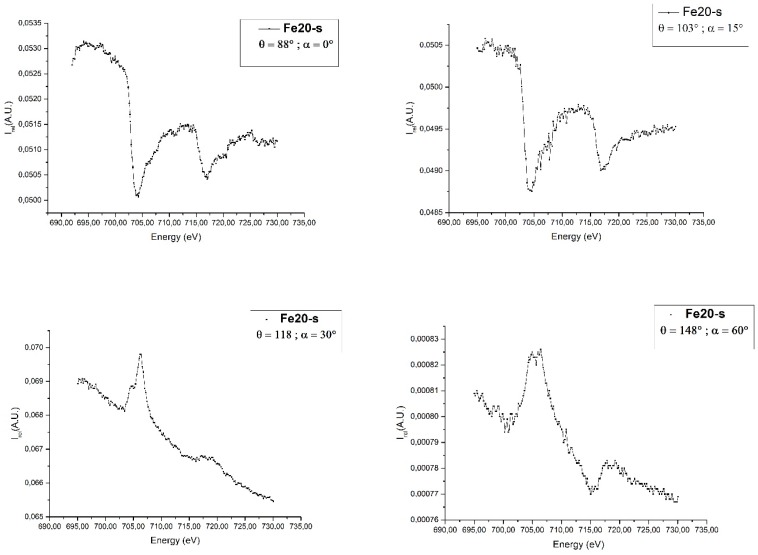
The four graphs above show the response of the CNTS-20Fe-s sample when the angle between the CNTSs axis and beam changes, using the total electron yield (TEY) technique; on y axis, the intensity is reported, it is in arbitrary units.

The presence of such a ‘nano-focusing’ effect means that measuring the L3 peak intensities alone does not provide reliable, quantitative information about the electronic properties from the single iron particles, as it is possible to notice in the two last graphs of Figure 7.

**Figure 8:**
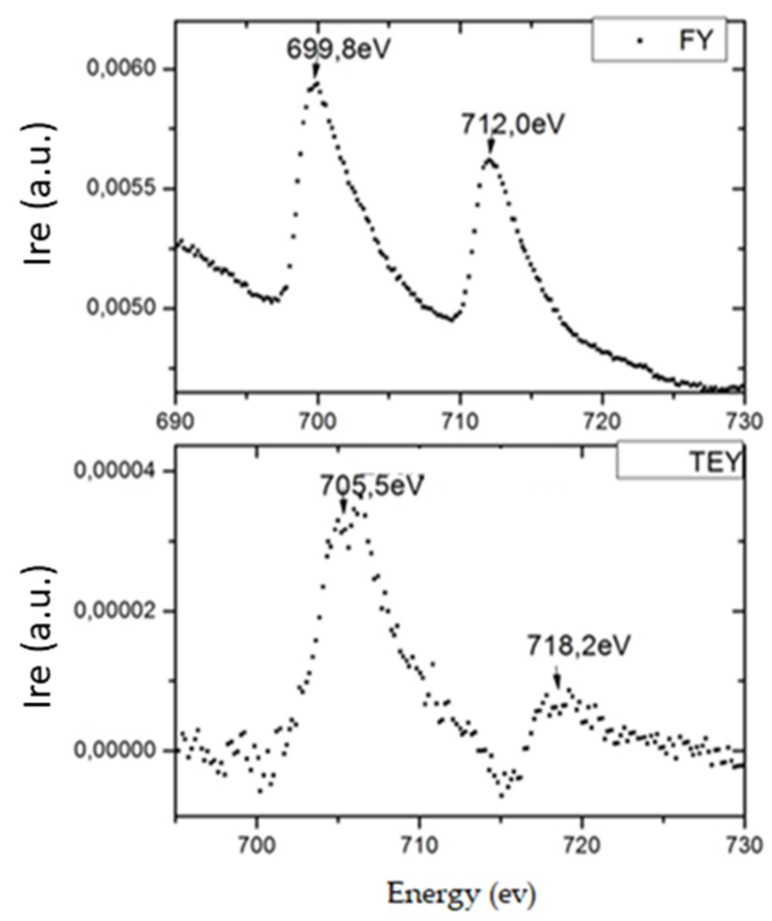
Shift of signal spectra between the fluorescence yield (FY) detector and TEY one, acquired using θ angle at 88° degrees; on y axis, the Intensity is reported, it is in arbitrary units.

In order to verify if this effect can be surface related (as TEY is sensitive roughly speaking to the first 10 nm of depth, at most) we repeated the analysis for the CNTS-20Fe-r and CNTS-20Fe-s samples, using Fluorescence Yield (FY) technique. This technique is sensitive to the photons coming from a much thicker layer of the sample, hence from a larger amount of iron. Moreover, the signals of spectra are at a lower energy than the signals registered with TEY (Figure 8): The difference is at least partly understood as the work function of iron in the photoelectric effect (4.5 eV) [29]. 

**Figure 9:**
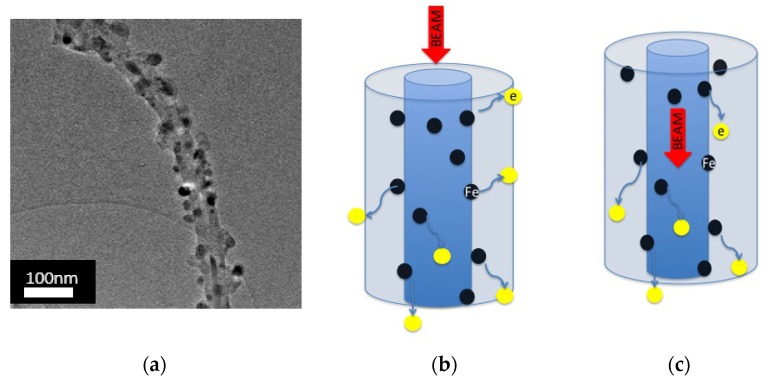
(**a**) Transmission Electron Microscope (TEM) image of CNTs and iron nanoparticles around the tube; (**b**) and (**c**) mechanism of electrons migration.

No evidence for inversion of the peaks was found by FY analysis’, in contrast with the occurrence when the same measurements were carried out by TEY. This difference between the two techniques outcomes, along with the angular behavior of the inversion peak in TEY, is a very interesting feature, worth further thorough investigation. It is reasonable that this behavior is due to strong coupling between the iron nanoparticles orbitals and the CNTs ones (Fe-C): When the incident beam is perpendicular to the sample surface (parallel to the CNTs axis), emitted electrons are captured by valence bands of CNTs and are able to move away to the grounded sample holder, Figure 9. This is a surface effect, restricted to a region a few nanometers deep.

**Figure 10:**
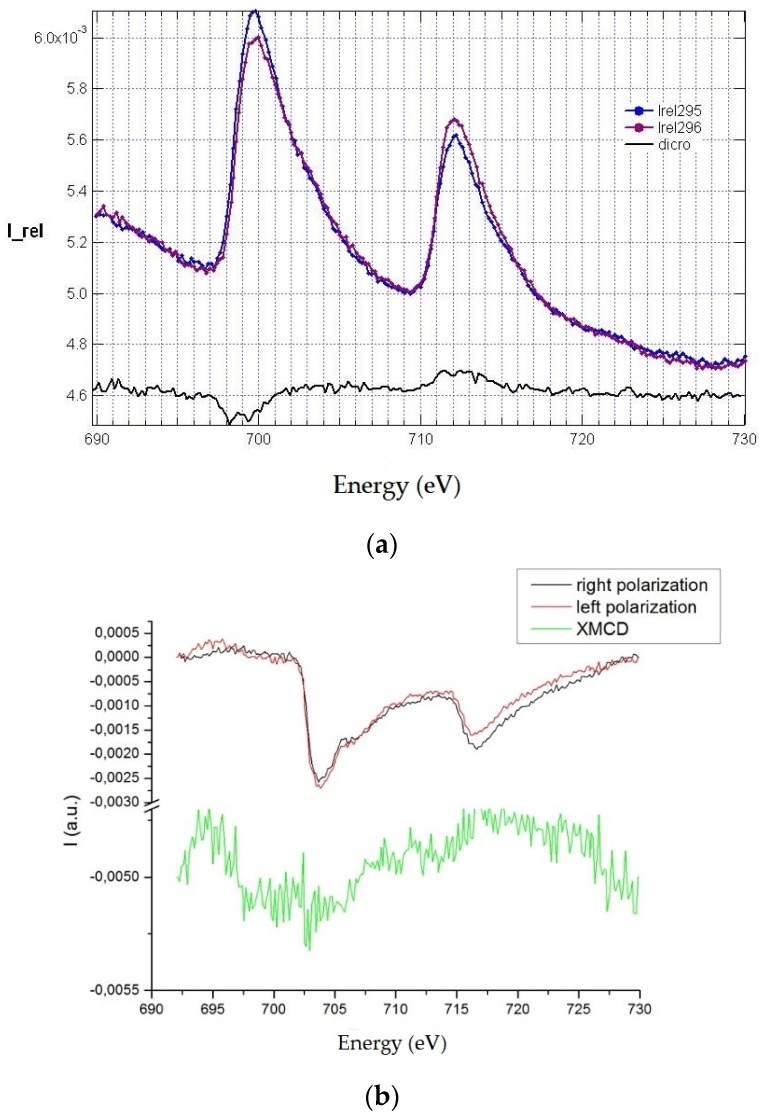
(**a**) The two recorded spectra showing a very clear dichroic signal (black solid line); on y axis, the intensity is reported, it is in arbitrary units; (**b**) circular dichroism for the samples of CNTs with iron at room temperature (Fe20-s); on y axis, the intensity is reported, it is in arbitrary units.

Using the FY technique, the scans in Figure 10a were carried out using two opposite circular polarizations in order to evidence circular dichroism. The difference between the two recorded spectra (Figure 10) shows a very clear dichroic signal (black solid line). 

Notwithstanding angular dependence of spectra registered with the TEY detector, circular dichroism is present in all cases, as Figure 10b shows.

It is clear, at this point, that angular dependence of TEY spectra are due to CNTSs effect and not to modifications in the electronic states of the iron nanoparticles.

## 4. Discussion

Studies on the L3,2 absorption edges of the iron (Fe) nanoparticles embedded in mainly oriented MWCNTs layers grown by the thermal CVD technique catalyzed by this transition metal, were carried out using XMCD spectroscopy in the soft X-ray regime by the synchrotron radiation. The X-rays kick some electrons out of the sample, making the latter slightly positively charged. Hence, some electron current flows towards the sample in order to balance this depletion. This current is exactly what gets measured in the TEY technique and what causes the above-mentioned upward bump (i.e., an increase of current). It is possible to underline, as Yueh et al. showed in reference [30], a transfer of charge from Fe to CNTs, which can also explain the presence of this current. The presence of a cluster of catalyst inside the tube were able to modify the Density of State of the CNTs. In this paper, the catalyst was present in the tube but in our sample, the TEM analysis showed the presence of iron clusters around the tubes, thus it possible to get more charge around the tubes [30]. Conversely, for the smooth surface, the current decreases at the iron L3,2 edges for some reason, which will require further careful investigation. The only information that we have at this stage is that the inversion takes places between 15° and 30° degrees. An unexpected result was found: The anomalous inversion of the absorption peaks. This peculiar behavior, which showed up with linear-normal polarization, seems to be quite interesting.

We wanted to try to repeat the measurements, at least some of them, on the CNTs-Fe by FY, since we wanted to find out whether the anomalous inversion of the absorption peaks would show up with this measuring technique too. We conjectured that no inversion would show up by this technique.

The overall picture can be drawn as follows. Because the tip growth mechanism is at work, the rough surface is richer in catalyst particles encapsulated in the CNT tips, while the smooth surface is richer in catalyst particles decorating the exterior of the CNT. As the XMCD sample has a very small depth, the signals coming from the two surfaces have different features. However, the presence of the encapsulated catalyst is limited to the uppermost region of the smooth surface. This means that when the FY technique is used in both cases, smooth as well as rough surfaces, most of the signal is provided by the catalyst particles externally decorating the CNTs.

Another feature that helps in understanding the results is that the region closer to the substrate, that gives rise to the smooth surface is characterized by a more ordered alignment of CNT (see Figure 6) with respect to the rough surface region. This emphasizes the electron mobility along the CNT surface as it limits the presence of defects and electron traps that are strictly related to kinks.

Finally, we highlighted the role of the difference in composition of the iron containing nanoparticles. The encapsulated nanoparticles are mainly composed by iron carbides [31] while the decorating nanoparticles are mainly iron oxides [32].

In summary, the different nature of the inner and outer iron nanoparticles together with the different sampling depth of the two techniques (XMCD and FY) and the difference in defectiveness close to the two surfaces are fully consistent with the experimental results.

## Figures and Tables

**Table 1:** This table shows the synthesis parameters of the samples containing iron.

Nickname Code	Iron Carrier/Carbon Carrier Ferrocene/Camphor	Synthesis Duration (minutes)	Temperature (°C)
CNTS-20Fe [FCCF158]	1/20	150	850
CNTS-30Fe [FCCF111]	1/30	70	850

## References

[1] Iijma S., Ichihashi T. (1993). Single-Shell Carbon Nanotubes of 1-nm Diameter. Nature.

[2] Li Z., Zhang L., Resasco D.E., Mun B.S., Requejo F.G. (2007). Angle-resolved x-ray absorption near edge structure study of vertically aligned single-walled carbon nanotubes. Appl. Phys. Lett..

[3] Stohr J. (1992). NEXAFS Spectroscopy.

[4] Roa D.B., Barcelos I.D., de Siervo A., Pirota K.R., Lacerda R.G., Magalhães-Paniago R. (2010). Observation of ferromagnetism in PdCo alloy nanoparticles encapsulated in carbon nanotubes. Appl. Phys. Lett..

[5] Mason W.R. (2007). A Practical Guide to Magnetic Circular Dichroism Spectroscopy.

[6] Caldwell D., Thorne J.M., Eyring H. (1971). Magnetic Circular Dichroism. Annu. Rev. Phys. Chem..

[7] Stephens P.J. (1974). Magnetic Circular Dichroism. Annu. Rev. Phys. Chem..

[8] Available online: http://www-ssrl.slac.stanford.edu/stohr/xmcd.htm

[9] Aharonov Y., Bohm D. (1959). Significance of electromagnetic potentials in the quantum theory. Phys. Rev..

[10] Goering E., Fuss A., Weber W., Will J., Schütz G. (2000). Element specific x-ray magnetic circular dichroism magnetization curves using total electron yield. J. Appl. Phys..

[11] Bellucci S., Onorato P., Shunin Y.N., Zhukovskii Y.F., Burlutskaya N. Multiwall carbon-nanotube interconnects: Radial effects on physical models and resistance calculations for various metal substrates. Proceedings of the 2010 International Semiconductor Conference (CAS).

[12] Gautama S., Thakur P., Augustine S., Kang J.K., Kim J.Y., Brookes N.B., Asokan K., Chae K.H. (2012). Electronic and magnetic structure of carbon nanotubes using X-ray absorption and magnetic circular dichroism spectroscopy. arXiv.

[13] Esquinazi P., Barzola-Quiquia J., Spemann D., Rothermel M., Ohldag H., García N., Setzer A., Butz T. (2010). Magnetic order in graphite: Experimental evidence, intrinsic and extrinsic difficulties. J. Magn. Magn. Mater..

[14] Musso S., Zanetti M., Giorcelli M., Tagliaferro A., Costa L. (2009). Gas chromatography study of reagent degradation during CVD carbon nanotube growth. J. Nanosci. Nanotechnol..

[15] Dai H. (2002). Carbon nanotubes: Opportunities and challenges. Surf. Sci..

[16] Dai H. (2002). Carbon nanotubes: Synthesis, integration, and properties. Acc. Chem. Res..

[17] Dai H., Kong J., Zhou C., Franklin N., Tombler T., Cassell A., Fan S., Chapline M. (1999). Controlled chemical routes to nanotube architectures, physics and devices. J. Phys. Chem..

[18] Jorio A., Dresselhaus G., Dresselhaus M.S. (2008). Carbon Nanotubes. Topics Applied Physics.

[19] Zaikovsky V.V., Chesnokov V.V., Buyanov R.A. (1999). Symmetric spiral patterns of filamentary carbon formed from butadiene-1,3 on the N—Cu/MgO catalyst. Regularities and mechanism of growing. Kinet. Catal..

[20] Pham-Huu C., Vieira R., Louis B., Carvalho A., Amadou J., Dintzer T., Ledoux M.J. (2006). About the octopus-like growth mechanism of carbon nanofibers over graphite supported nickel catalyst. J. Catal..

[21] Chiang W.-H., Sankaran R.M. (2009). Linking catalyst composition to chirality distributions of as-grown single-walled carbon nanotubes by tuning NixFe_1−x_ nanoparticles. Nat. Mater..

[22] Porro S., Musso S., Giorcelli M., Tagliaferro A. (2006). Thermal CVD growth of Carbon Nanotubes thick layers. Adv. Sci. Technol..

[23] Taurino I., Carrara S., Giorcelli M., Tagliaferro A., De Micheli G. (2012). Comparison of two different carbon nanotube-based surfaces with respect to potassium ferricyanide electrochemistry. Surf. Sci..

[24] Musso S., Porro S., Giorcelli M., Chiodoni A., Ricciardi C., Tagliaferro A. (2007). Macroscopic growth of carbon nanotube mats and their mechanical properties. Carbon.

[25] Available online: www.elettra.trieste.it/elettra-beamlines/bach.html

[26] Briones-Leon A., Ayala P., Liu X., Kataura H., Yanagi K., Weschke E., Eisterer M., Pichler T., Shiozawa H. (2013). Orbital and spin magnetic moments of transforming one-dimensional iron inside metallic and semiconducting carbon nanotubes. Phys. Rev. B.

[27] Rodrìguez A.F., Kleibert A., Bansmann J., Nolting F.J. (2014). In situ magnetic and electronic investigation of the early stage oxidation of Fe nanoparticles using X-ray photo-emission electron microscopy. Phys. Chem. Chem. Phys..

[28] Okotrub A., Kanygin M.A., Sedelnikova O.V., Gusel’nikov A.V., Belavin V.V., Kotosonov A.S., Bulusheva L. (2010). Interaction of ultrasoft X-rays with arrays of aligned carbon nanotubes. J. Nanophotonics.

[29] Tipler P.A., Llewellyn R.A. (2012). Modern Physics.

[30] Yueh C.L., Jan J.C., Chiou J.W., Pong W.F., Tsai M.H., Chang Y.K., Chen Y.Y., Lee Y.F., Tseng P.K., Wei S.L. (2001). Electronic structure of the Fe-layer-catalyzed carbon nanotubes studied by x-ray-absorption spectroscopy. Appl. Phys. Lett..

[31] Pellegrino L., Daghetta M., Pelosato R., Citterio A., Mazzocchia C.V. (2013). Searching for Rate Determining Step of CNT Formation: The Role of Cementite. Chem. Eng. Trans..

[32] Cao H., Zhu M., Li Y. (2006). Decoration of carbon nanotubes with iron oxide. J. Solid State Chem..

